# Comparative Study of Perioperative Outcomes Between Modified Blumgart Duct to Mucosa and Dunking Pancreaticojejunostomy

**DOI:** 10.7759/cureus.34418

**Published:** 2023-01-30

**Authors:** Krishna M Adhikari, Deepak Sharma, Romi Dahal, Bishnu P Kandel, Paleswan J Lakhey

**Affiliations:** 1 Department of Surgical Gastroenterology, Maharajgunj Medical Campus, Institute of Medicine, Tribhuvan University Teaching Hospital, Kathmandu, NPL

**Keywords:** duct to mucosa, dunking, modified blumgart, perioperative outcomes, hepato-pancreato-biliary surgery

## Abstract

Background: Pancreaticojejunostomy (PJ) is the ''Achilles heel” of pancreaticoduodenectomy (PD) which affects perioperative as well as oncological outcomes. However, there is a lack of information about the superiority of the type of anastomosis in terms of overall morbidity and postoperative pancreatic fistula (POPF) after PD. Here, we compare the outcomes of modified Blumgart PJ with the dunking technique of PJ.

Methodology: A case-control study of a prospectively maintained database of 25 consecutive patients undergoing modified Blumgart PJ (study group) and 25 patients who underwent continuous dunking PJ (control group) between January 2018 to April 2021 was done. Between groups, comparisons were made for the duration of surgery, intraoperative blood loss, original fistula risk score, overall complications as graded by Clavien Dindo (CD), POPF, post pancreatectomy haemorrhage (PPH), delayed gastric emptying (DGE), and 30-day mortality at 95% confidence level.

Results: Among 50 patients, 30 (60%) were male. The most common indication for PD was ampullary carcinoma (44% in the study group vs. 60% in the control group). The duration of surgery was approximately 41 minutes longer in the study group compared to the control (p = 0.02), while the intraoperative blood loss was similar between the two groups (496.00 ± 226.35 ml vs 508.00 ± 180.67 ml, p = 0.84). While there was no significant difference in mean fistula risk score between the two groups, the POPF (8% vs 32%, p = 0.03), PPH (0% vs 20%, p =0.02), and overall major complications (CD≥ III) according to CD Grading (12% vs 40%, p = 0.02) were significantly lower in the study group. Similarly, the duration of hospital stay in the study group was 4.64 days shorter than the control group (p = 0.001). However, there was no significant difference in the 30-day mortality between the two groups.

Conclusions: Modified Blumgart pancreaticojejunostomy has better perioperative outcomes in terms of procedure-specific complications like POPF, PPH, overall major postoperative complications, and duration of hospital stay.

## Introduction

Pancreaticoduodenectomy (PD) is a complex procedure in the surgical management of malignant and benign tumours of the periampullary regions. The outcome of this procedure, however, is affected by pancreatoenterostomy, the Achilles heel after PD, which has a higher chance of surgical complications potentially leading to postoperative pancreatic fistula (POPF) [1].

While improvements in pancreatic resection procedures as well as advancement in postsurgical ICU care and intervention radiology procedure has decreased mortality to less than 5% [2], many studies still report high morbidity rates ranging from 30% to 50% even in high-volume centers [3,4]. Many complications like POPF, postpancreatectomy hemorrhage (PPH), delayed gastric emptying (DGE), and infection are associated with PD [5]. More importantly, a POPF with leakage of pancreatic juice into the intra-abdominal region remains a potentially fatal condition. Reintervention may be necessary to minimize the risk of mortality due to POPF-associated sepsis and/or intraluminal or extraluminal hemorrhage [6].

The historic focus of most research in pancreatic surgery has been on understanding and identifying the causes and risk factors and implementing preventive measures to mitigate POPF [7]. Different anastomotic techniques for pancreaticojejunostomy (PJ) have been used to mitigate POPF. While some recent studies have highlighted minimized complication rate and POPF in the Blumgart procedure compared to dunking invagination PJ [8], others have documented no such benefit of the Blumgart procedure in reducing POPF incidence [9]. Nevertheless, efforts to decrease postoperative mortality and morbidity should focus on evaluating the efficacy of various PJ techniques in reducing POPF and PPH. Using a prospectively maintained database of patients, here we compare the perioperative outcomes of dunking PJ and modified Blumgart PJ techniques at our centre.

This article was previously presented as a poster presentation at the 32nd national conference of the Indian Association of Surgical Gastroenterology (IASGCON 2022, on 15th October 2022).

## Materials and methods

Study design

This retrospective study uses a prospectively maintained database of patients who underwent PD from 2018 to 2021 at a hospital in Nepal. The initial 25 consecutive patients during the study period who underwent continuous dunking PJ were considered the control group, while the latter 25 consecutive patients who underwent modified Blumgart PJ were considered the study group. Ethical approval to conduct the study was obtained from the Institutional Review Committee of the Institute of Medicine, Tribhuvan University (129/6-11 E2).

The demographics, intraoperative variables (e.g., the texture of pancreatic parenchyma, pancreatic duct diameter, pathology, intraoperative blood loss, fistula risk score), and postoperative variables (e.g., procedure-specific complications, length of stay, histopathology) were retrieved and analyzed. Histopathology reports of surgical specimens were retrieved from the Pathology Department database system. Follow up of patients were done in the outpatient department and by telephone questionnaire for three months at the interval of 30 days.

An original fistula risk score was calculated by taking perioperative and postoperative parameters like the texture of the pancreatic gland, pathology, the diameter of the main pancreatic duct, and the amount of intraoperative blood loss [10].

Preoperative biliary drainage was done on patients who were on cholangitis or if the surgical procedure couldn't be done within 10 days of hospital admission due to the unavailability of an operating theater slot. For those patients who had undergone preoperative biliary drainage definitive surgery was done after four weeks.

Surgical procedure

Modified Blumgart PJ 

**Figure 1:**
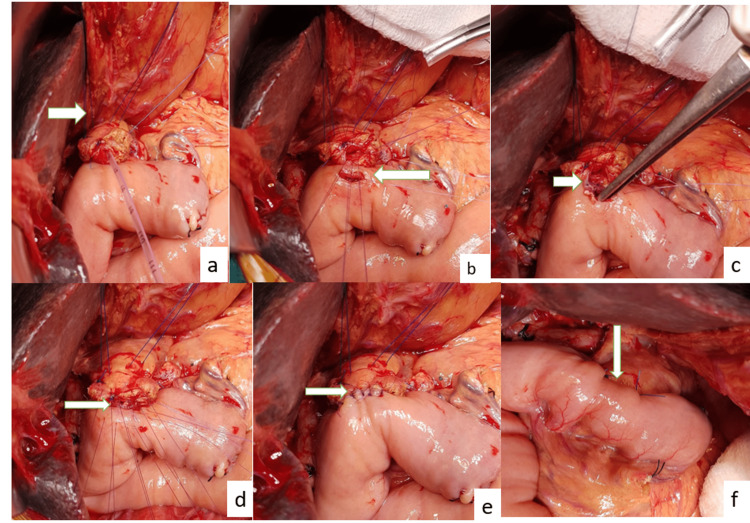
Modified Blumgart Pancreaticojejunostomy a) trans pancreatic U suture along with serosa of jejunum taken with 3-0 polypropylene b) enterostomy and posterior layer of pancreatic duct to mucosa with 5-0 polydioxanone c) completion of the posterior anastomosis d) anterior layer of duct to mucosa with 5-0 polydioxanone suture e) completion of the anterior anastomosis f) completion of anterior layer of U stitch

In the modified Blumgart anastomosis, three or more trans-pancreatic U-stitches of 3-0 polypropylene suture through the pancreas, then the seromuscular layer of the jejunum, and again through the pancreas were taken. Usually, one U stitch was placed above the pancreatic duct and two below the pancreatic duct. A duct to mucosa anastomosis was done with size 5-0 polydioxanone suture at 12, 2, 4, 6, 8, and 10 o’clock positions of the pancreatic duct and jejunum. The previous suture of the U stitch was used to take the bite of the seromuscular layer of the jejunum and then tied at the antimesenteric surface of the jejunum to fully cover the pancreatic stump with jejunal serosa (Figure 1) [11].

Dunking Method

**Figure 2:**
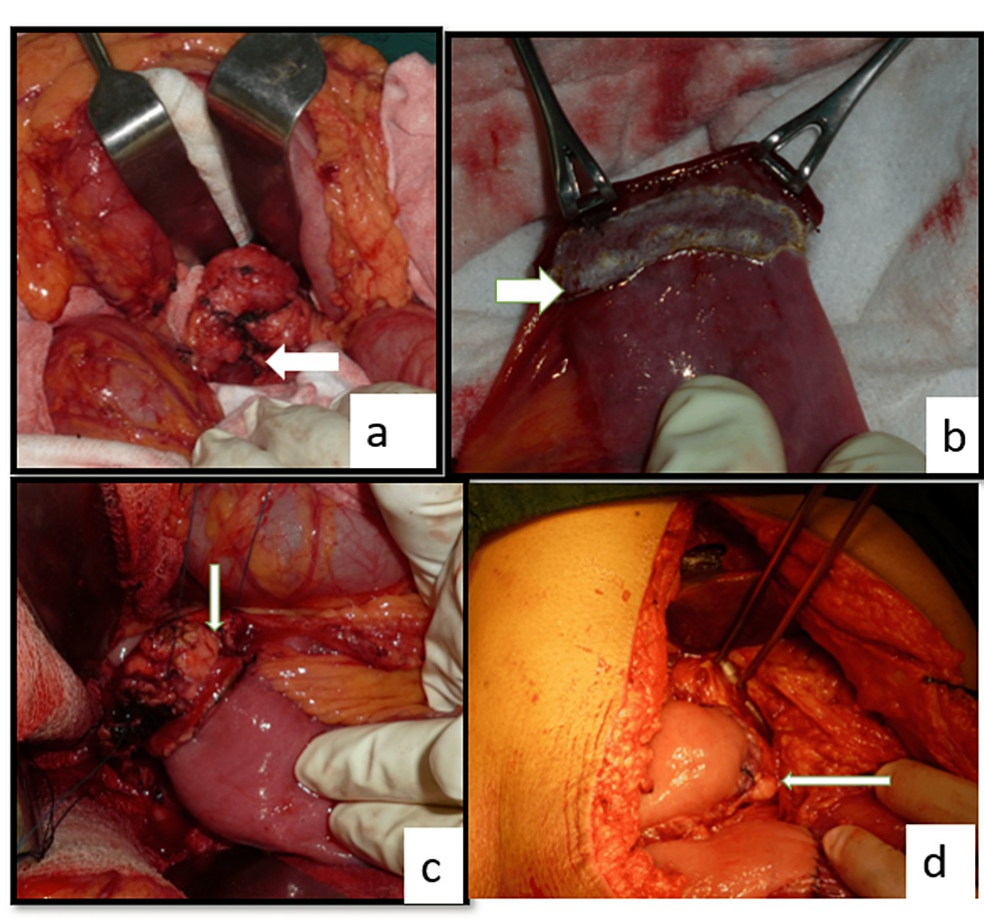
Dunking Pancreaticojejunostomy a: mobilisation of pancreas b: preparation of jejunal limb c: posterior continuous suture d: anterior suture after dunking the pancreatic stump

After specimen removal, the pancreas was mobilized from the retroperitoneum for at least 2-3 cm, and stay sutures at the superior and inferior border of the pancreatic remnant were taken with 3-0 silk. Then a loop of jejunum was brought up to the region of the cut surface of the pancreas and the staple line applied during transection of the jejunum was excised. By using a 3-0 polypropylene suture and starting at the upper border of the pancreas as far distally as mobilized, horizontal mattress sutures taking the pancreas capsule on one side and the serosa of the edge of the jejunum were taken to complete the posterior layer of anastomosis. Invagination of the pancreatic stump to the end jejunal limb was done with a previously placed stay suture that was passed through the lumen of the jejunum, followed by the completion of the anterior layer of anastomosis. This procedure invaginates or ‘dunks’ the whole of the cut edges of the pancreas into the jejunal lumen to allow the apposition of the pancreatic capsule to the jejunal mucosa (Figure 2) [12].

Postoperative complication grading and statistical analysis

Postoperative complications after PD were POPF, PPH, and DGE. POPF was defined and graded according to the International Study Group on Pancreatic Surgery (ISGPS) 2016 [13]. PPH was defined and graded based on the onset, location, and severity of bleeding following the ISGPS 2007 definition [14]. Similarly, DGE was identified and graded using the ISGPS 2007 definition [15]. Postoperative pancreatic leakage (e.g., intra-abdominal abscess/fluid collection) was detected by computed tomography (CT) scans. The non-procedure-specific complication was defined and graded according to the Clavien Dindo (CD) score and scores III or higher were recorded and analyzed.

Statistical comparisons between the two groups were done using the student t-test for continuous variables and the chi-square test for the categorical variables. All statistical tests were conducted at a 95% confidence level using SPSS Statistics for Windows, version 25.0 (IBM Corp., Armonk, NY, USA).

## Results

The clinical and demographic characteristics of patients in the control group (25 patients who underwent continuous dunking PJ) were not different from the study group (25 patients who underwent modified Blumgart PJ) (Table 1). Mean age, gender, indications for PD, preoperative biliary drainage, and fistula risk score were similar between the two groups. Ampullary carcinoma was the dominant indication for PD in about 44% of patients in the study group and 60% in the control group. As such, preoperative biliary drainage was done in about 32% of patients in the study group and 24% in the control group. Among patients with preoperative biliary drainage, 75% in the study group and 67% in the control group had percutaneous transhepatic biliary drainage (PTBD). The mean fistula risk score was 3.4 for both groups suggesting that factors predicting POPF were similar between groups.

**Table 1:** Clinical and demographic characteristics of patients PD- Pancreaticoduodenectomy ERCP- Endoscopic retrograde cholangiopancreatography PTBD- Percutaneous transhepatic biliary drainage NA: not applicable

Variables	Modified Blumgart (n=25)	Continuous Dunking (n=25)	p-value
Age in years, mean±SD	59.04 ± 10.98	57.64 ± 12.84	0.68
Male, n (%)	16 (64)	14 (56)	0.56
Indication for PD, n (%)			0.37
Ampullary carcinoma	11 (44)	15 (60)	
Distal cholangiocarcinoma	6 (24)	2 (8)	
Duodenal carcinoma	0 (0)	1 (4)	
Pancreatic carcinoma	7 (28)	5 (20)	
Others	1 (4)	2 (8)	
Preoperative biliary drainage, n (%)			0.77
No	17 (68)	19 (76)	
Yes	8 (32)	6 (24)	
Methods of drainage			NA
ERCP	2 (25)	2(33.3)	
PTBD	6 (75)	4 (66.7)	
Fistula Risk score, mean±SD	3.4±1.71	3.4±1.6	0.1

Between the two groups, there was no difference in perioperative variables like pancreatic texture, main pancreatic duct diameter, intraoperative blood loss, and procedure-specific complications like DGE (Table 2). Similarly, none of the patients in the study group required venous resection but in the control group, two (8%) patients required venous resection. Only two (8%) patients exhibited clinically relevant POPF following the modified Blumgart PJ compared to about eight (32%) following the continuous dunking PJ. Likewise, there was no incidence of PPH among patients following the modified Blumgart PJ compared to the five (20%) incidences in patients after the continuous dunking PJ. The duration of surgery was 41 minutes longer in the modified Blumgart group (Table 2; p=0.02).

**Table 2:** Perioperative variables and procedure-specific complications as affected by PJ procedure POPF: postoperative pancreatic fistula PPH: postpancreatectomy hemorrhage DGE: delayed gastric emptying

Variable	Modified Blumgart (n:25)	Continuous Dunking (n: 25)	P value
Pancreatic texture, n (%)			0.08
Soft	12(48)	18(72)	
Firm	13(52)	7(28)	
Main pancreatic duct diameter, n (%)			0.15
< 5mm	13(52)	18(72)	
≥5mm	12(48)	7(28)	
Venous resection, n (%)			0.11
No	25 (100)	23(92)	
Yes	0 (0)	2(8)	
Intraoperative blood loss in ml, mean±SD	496.00 ± 226.35	508.00 ± 180.67	0.84
Duration of Surgery in minutes, mean±SD	410.80 ± 59.01	369.60 ± 57.77	0.02
POPF, n (%)			0.03
No	23(92)	17(68)	
Yes	2(8)	8(32)	
PPH, n (%)			0.02
No	25(100)	20(80)	
Yes	0(0)	5(20)	
DGE, n (%)			1.0
No	23(92)	23(92)	
Yes	2(8)	2(8)	

In the control group, there were three patients (12%) who had 30-day mortality due to procedure-specific complications and 40% of patients had major postoperative complications. On the other hand, in the study group, there was no 30-day mortality and only three (12%) patients had major postoperative complications reducing the length of hospital stay by 4.6 days (Table 3).

**Table 3:** Overall complications

Variables	Modified Blumgart N (25)	Continuous dunking N (25)	p-value
30-day mortality, n (%)	0 (0)	3 (12)	0.07
Clavien Dindo grading, n (%)			0.02
< 3	22 (88)	15 (60)	
≥3	3 (12)	10 (40)	
Length of hospital stay in days, mean ±SD	11.64 ± 2.08	16.28 ± 5.72	0.001

## Discussion

While recent advances in surgical procedures as well as advancements in postsurgical ICU care and interventional radiological procedures have improved perioperative outcomes and have greatly reduced mortality after PD, several challenges exist in bringing morbidity rates down [2]. This can be attributed to procedure-specific postoperative complications like POPF, PPH, and DGE. Because POPF is a primary cause of morbidity after PD, researchers have put significant effort into developing and refining surgical techniques for PJ healing over the past several decades [7]. These techniques focus on completely securing the gland to the jejunum [9]. However, the availability of multiple variations of PJ techniques requires comparative studies to identify best practices that minimize risks, reduce mortality, and morbidity, and improve safety and patient outcomes. To that end, our retrospective study provides comparative insights into peri- and postoperative outcomes of two common PJ techniques: modified Blumgart and continuous dunking PJ.

Despite several types of pancreaticoenterostomies, a literature review by Strobel et al. [2] indicated that POPF rates are rarely affected by the location of pancreatic anastomosis (e.g., jejunum or stomach) or suturing techniques, and stenting (e.g., internal or external) method for anastomosis. For example, Casadei et al. [8] did not observe significant differences in clinically recognizable POPF rates between the Blumgart technique and invagination (or dunking) PJ. Unlike these studies, our study provides evidence that POPF rates can be significantly reduced using the modified Blumgart technique instead of invagination (or dunking) PJ. Grobmyer et al. [16] suggested that Blumgart PJ can be performed on all patients with identifiable pancreatic ducts, and is associated with significantly low postoperative morbidity and mortality. We started doing modified Blumgart PJ in 2020. In the small cohort of 25 consecutive cases, POPF decreased from 32% to 8% and PPH from 20% to 0%. We attribute these results to the use of a smaller number of sutures that decreases shearing and tearing forces that contribute to the incidence of POPF. It has been suggested that modified Blumgart PJ has a tamponade effect that helps to secure the hemostasis and decrease the incidence of PPH. Likewise, the reduction of POPF incidence in the modified Blumgart anastomosis group has been attributed in the literature to the use of a few sutures (one to three) to minimize tangential tension and shear forces negatively influencing the healing of POPF [10-11]. A relative decrease in the rate of mortality and the duration of hospital stay in the modified Blumgart PJ of our study was presumably due to a decrease in POPF and PPH incidence.

Various risk scores have been used to predict clinically relevant POPF after PD [10,17]. The original fistula risk score used in this study takes into account the pancreatic texture, pathology, pancreatic duct diameter, and intraoperative blood loss [10]. Although the mean fistula score (3.4 ± 1.71 vs 3.4 ± 1.6) was similar between groups, POPF was significantly high in the dunking group.

Most of the limitations of our study are inherent in studies that are retrospective in nature and conducted at a single center [18]. Specifically, the sample size in our study was small (n=50, 25 in each group) and interventions were applied at different times. Gain in surgical experience over time among surgeons conducting PJ has presumably introduced some bias in the success of PJ outcomes in our study. Nevertheless, statistically significant differences between groups underscore the importance of the modified Blumgart technique over dunking PJ for reducing clinically relevant POPF and surgical mortality.

## Conclusions

The modified Blumgart PJ has better perioperative outcomes than continuous dunking PJ in terms of procedure-specific complications like POPF, PPH, overall major postoperative complications, and duration of hospital stay. Furthermore, this study not only provides preliminary insights into the benefits of modified Blumgart PJ but also underscores the need for robust randomized control trials to evaluate the relevance of this technique over other contemporary approaches (e.g., invagination, duct to mucosa) in reducing POPF and associated mortality.
